# Exploring Two Protocols of FISH Using Cytocell SYT-SSX Probe on Formalin Fixed Paraffin Embedded Tissue Sections

**DOI:** 10.31557/APJCP.2020.21.5.1241

**Published:** 2020-05

**Authors:** Aidy Irman Yajid, Siti Norasikin Mohd Nafi, Nor Aziah Salehan, Sharifah Emilia Tuan Sharif

**Affiliations:** 1 *Department of Pathology, School of Medical Sciences, Universiti Sains Malaysia, Health Campus, Kota Bharu, Kelantan, Malaysia. *; 2 *Department of Pathology, Hospital Universiti Sains Malaysia, Kota Bharu, Kelantan, Malaysia. *; 3 *Department of Pathology, Hospital Queen Elizabeth, Kota Kinabalu, Sabah, Malaysia. *

**Keywords:** SYT, SSX, fish, synovial sarcoma, cytocell

## Abstract

**Background:**

Chromosomal translocation t(X;18)(p11.2;q11.2) is the cytogenetic hallmark of synovial sarcoma and have been identified as an alternative diagnostic strategy in differentiating synovial sarcoma from other histologic mimics. This study was carried out to test the efficacy of two FISH protocols using the SYT-SSX break apart probe from Cytocell.

**Methodology:**

Representative paraffin blocks of synovial sarcoma were utilized in this study. FISH study was performed on formalin-fixed paraffin embedded tissue sections using the SYT-SSX break apart probe from Cytocell, to detect two form of SYT-SSX transcript, SYT-SSX1 and SYT-SSX2. FISH protocol, including the hybridization was done following two different protocols, Cytocell FISH protocol and Optimized Dako FISH protocol.

**Results:**

Tissue samples subjected to FISH using Cytocell FISH protocol showed the absence of signal corresponding to the probe used. Utilizing Optimized Dako FISH protocol, the two signals (red and green) corresponding to the break-apart probes was detected. These findings suggested that Optimised Dako FISH protocol is more suited for use with the tested probe on paraffin embedded tissues in comparison to Cytocell FISH protocol.

**Conclusion:**

Optimised Dako FISH protocol was noted to be more suited for detecting SYT-SSX FISH signals on paraffin embedded tissues in comparison to Cytocell FISH protocol.

## Introduction

Synovial sarcoma (SS) is one of the soft tissue tumour which greatly benefited from the molecular advancement. Synovial sarcoma is classified as group of soft tissue tumours of uncertain differentiation under the recent WHO classification (Christopher et al., 2013). Despite its name, it is extremely uncommon in joint cavities but arises in areas with no apparent relation to the synovial structure (Goldblum et al., 2013). This tumour commonly leads to diagnostic difficulties due to its morphological features and mimics many benign and malignant spindle cell tumours. The unique features of SS is that it is a high grade sarcoma but lacking of nuclear pleomorphism and hyperchromatism which renders the difficulty in the diagnosis especially in monophasic and poorly differentiated SS. In addition, TLE-1 immunohistochemistry which is said to be a highly sensitive and specific to SS could also be expressed in small subset of other soft tissue tumours (Ikmal Hisyam Bakrin et al., 2016).

The molecular tools has given a better understanding of synovial sarcoma biology. It was shown that the presence of translocation t(X;18)(p11.2;q11.2) is considered a cytogenetic hallmark of synovial sarcoma, as it is present in approximately 90 to 95% of cases of synovial carcinoma (Minami et al., 2014). This translocation is useful as a specific marker for the differentiation of synovial sarcoma from malignant solitary fibrous tumor, spindle epithelial tumor with thymus-like differentiation and skin adnexal tumors (Argani et al., 1998). We thus carried out the investigation to determine the incidence of t(X;18) translocations in synovial sarcoma cases. 

The fusion transcripts (SS18-SSX1 and SS18-SSX2) can be detected by Fluorescence in situ Hybridization (FISH). This molecular test is very useful in solving diagnostic dilemmas, such as cases occurring at unexpected sites, or when immunohistochemical profile is inconclusive for diagnosis (Chuang et al., 2013). Thus, FISH remains the gold standard test in certain cancer diagnosis in view of its high sensitivity and specificity. For synovial sarcoma, the sensitivity and specificity of FISH for known synovial sarcoma was shown to be 96.7%, and 100% respectively (Arumugam et al., 2016). However, there were no reported studies that suggested the sensitivity or specificity of FISH in detecting subtypes of synovial sarcoma. Regardless, the ability of FISH to detect SYT-SSX fusion transcript allows us to determine the histological subtypes and the probable prognosis in synovial sarcoma patients. 

Utilizing SYT probe, it is possible to determine the translocation of SYT-SSX into the two forms of SYT-SSX fusion transcript; SYT-SSX1 and SYT-SSX2, which can also be identified using RT-PCR (Tvrdik et al., 2005). A study by Kawai et al., (1998) found a significant correlation between SYT-SSX gene and histologic subtype of tumors, in which the tumor containing SYT-SSX1 are biphasic and SYT-SSX2 are monophasic subtypes. These fusion transcripts appear to influence the histologic subtypes of synovial sarcoma. 

Due to the capability of FISH to detect these fusion transcripts of synovial sarcoma, as well as the absence of studies that utilizes SYT-SSX probe from Cytocell on paraffin embedded tissues, we carried out a study to test two different FISH protocol using the SYT-SSX break apart probe from Cytocell to determine which protocol is more suited to be used on paraffin embedded tissue. 

## Materials and Methods

*Synovial sarcoma specimens*

This study was conducted as a preliminary experiment to test the efficacy of two FISH protocols carried out at Hospital Universiti Sains Malaysia from a period of 12 months from July 2016 to July 2017. Ethical clearance for the study was obtained from the Ethical Committee, Universiti Sains Malaysia (JEPEM Code: USM/JEPeM/16050189). A total of 27 cases diagnosed histologically as synovial sarcoma (SS), were retrieved from registry book and computerized registry data files were selected. Representative paraffin block of the tumors was utilized in this study. 

*Histology assessment*

The tissue slides were deparaffinized and hydrated using standard procedures and stained with hematoxylin and eosin (H&E). The slides were viewed using Olympus BX43 light microscopy to identify the optimal region of the tumor.

*Fluorescent in situ hybridization (FISH)*

The Cytocell FISH protocol was carried out based on the Cytocell SYT Breakapart probe protocol (Cytocell, United Kingdom) as instructed in the user manual pamphlet. The Optimised Dako FISH protocol was conducted using modified Dako Histology FISH Accessory Kit (Agilent, United States). The SYT-SSX break apart probe from Cytocell was used in both Cytocell FISH protocol and Optimised Dako FISH protocol. The procedure for Cytocell FISH protocol and Optimised Dako FISH protocol is summarized in Table 1. 

**Figure 1:**
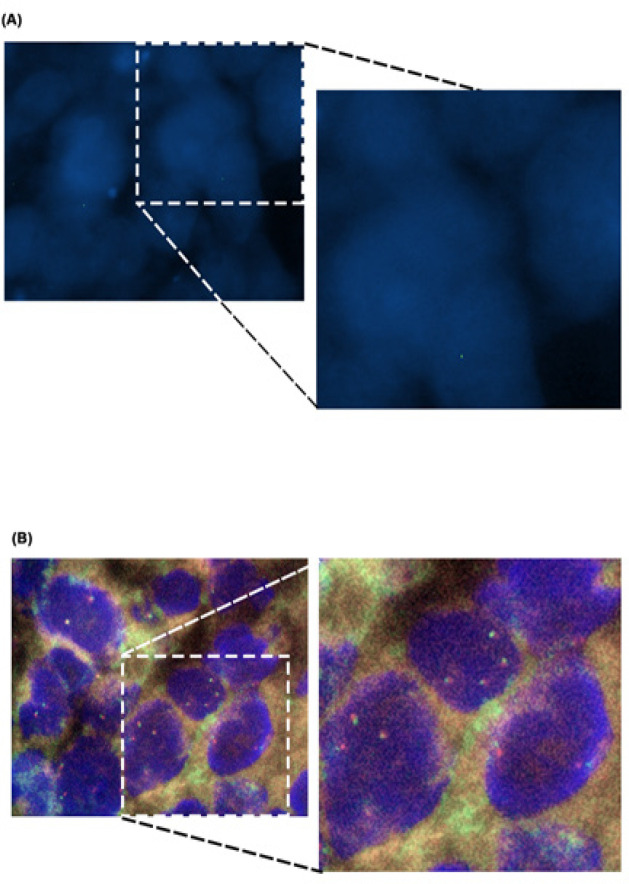
SYT-SSX FISH Analysis in Paraffin Embedded Tissue. (A) represents tissues that have been processed through Optimised Dako FISH protocol and (B) represents tissue that have been processed through Cytocell FISH protocol. Magnification x100, n=3

**Table 1:** Step by Step Procedure for FISH Cytocell FISH Protocol and Optimised Dako FISH Protocol

	Cytocell FISH protocol	Optimised Dako FISH protocol
Tissue slide and reagent preparation	The slide is placed on the hot plate at 60ᵒC for 1 hour.	Tissue slide was incubated at 37ᵒC overnight Pre-treatment solution is incubated at 97ᵒC 5 hours prior to tissue treatment. Tissue slide was placed on the hotplate at 60ᵒC for 30 minutes
Deparaffinization and rehydration	1. Xylene - 5 min 2. Xylene - 5 min 3. Alcohol 100% - 3 min 4. Alcohol 100% - 3 min 5. Alcohol 80% - 3 min 6. Alcohol 70% - 3 min 7. Wash Buffer - 3 min 8. Wash Buffer - 3 min	1. Xylene - 5 min 2. Xylene - 5 min 3. Alcohol 100% - 2 min 4. Alcohol 95% - 2 min 5. Alcohol 80% - 2 min 6. Alcohol 70% - 2 min 7. Wash Buffer - 3 min 8. Wash Buffer - 3 min
Pre-treatment	1. Pre-treatment solution were heated to 98°C prior to slide incubation. 2. Slides were incubated with the pre-treatment solution at 98ᵒC for 20 minutes 3. Slides were removed from the pre-treatment solution and were allowed to cool for 15 minutes at room temperature. 4. Slides were washed in PBS for twice for 3 minutes at room temperature.	1. Slides were incubated in pre-treatment solution that has been pre-heated (for 5 hours) at 97°C for 30 minutes 2. Slides were removed from the pre-treatment solution and were allowed to cool for 15 minutes at room temperature. 3. Slides were washed in wash buffer twice for 3 minutes at room temperature.
Enzyme digestion	1. Slides were tapped and wiped to remove excess PBS 2. A total of 150 µl of enzyme reagent were transferred onto the tissue and incubated for 15 minutes at room temperature. 3. The slides were washed in PBS at room temperature three times for 2 minutes 4. Slides were dehydrated in a series of 70%, 85%,95 & 100% alcohol for 2 minutes each at room temperature. 5. The slides were air dried for 10 minutes at room temperature.	1. Slides were tapped and wiped to remove excess wash buffer 2. The slides were placed in a 37ᵒC incubator followed by incubation with a drop of cold RTU pepsin that covers the tissue on the slides (depends on tissue size) for 1 minute. 3. Slides were washed in wash buffer twice for 3 minutes at room temperature. 4. Slides were dehydrated in a series of 70%, 80%,100% & 100% alcohol for 2 minutes each at room temperature. 5. Slides were air dried for 10 minutes at room temperature.
Probe application	1. Slides and probe were pre-warmed at 37ᵒC for 5 minutes. 2. A total of 15 µl of probe was applied onto the tissue followed by the application of coverslip and rubber sealant.	1. A total of 1.5µl - 5µl of probe is applied to the selected area on the tissue section (for 10mm-16mm in diameter coverslip). 2. Coverslip was applied, and the tissue and probe were sealed coverslip sealant.
Denaturation and hybridization	1. Slides were placed into the hybridizer (Agilent, United Sates) that was programmed to conduct 5 minutes of denaturation step at 75°C and overnight hybridization at 37°C.	1. Slides were placed into the hybridizer (Agilent, United States) that was programmed to conduct 5 minutes of denaturation step at 82°C and overnight hybridization at 45°C.
Stringent wash	1. Slides were incubated in stringent wash buffer 72°C for 2 minutes. 2. Slides were incubated in wash buffer at room temperature for 30 seconds prior to drainage of excess liquid. 3. The slides were subjected to a series of w minutes ethanol washes series at 70%,80% and 100% followed by the drainage of the slides.	1. Slides were immersed in stringent wash buffer at room temperature for a short rinse. 2. Slides were incubated in pre-heated (for 5 hours at 65ᵒC) stringent wash buffer for 10 minutes. 3. Following stringent wash, slides were washed with normal wash buffer twice for 3 minutes at room temperature. 4. The slides were dehydrated by a series of 2 minutes incubations in 70%,80% and 100% ethanol. 5. Slides were drained.
Mounting	1. A total of 15µl DAPI (brand) were applied on the tissue and the tissue was covered with coverslip. 2. The slides were incubated in the dark for 10 minutes at room temperature prior to analysis. the color to develop in the dark for 10 minutes.	1. A total of 10µl of fluorescence mounting medium containing DAPI was applied to the target area of slides and covered with coverslip. 2. Nail polish was applied to the edge of the coverslip to prevent the coverslip from moving. 3. Slides were placed in darkness for 10 minutes at room temperature prior to analysis.

## Results

Both FISH protocols was shown here to enable the detection of the nucleus of the cells. However, the utilization of Optimised Dako FISH protocol was shown to result in better and crispier nuclear stains in comparison to nucleus from samples stained using Cytocell FISH protocol (Figure 1). This observation suggests that although both protocols allowed for the detection of the cell nucleus, the steps described in the Optimised Dako FISH protocols allowed for better probe performance that led to better nuclear stain. 

Tissue samples subjected to FISH using the Cytocell FISH protocol showed the absence of signal corresponding to the probe used as shown in Figure 1 (A). Utilizing the Optimised Dako FISH protocol, we managed to achieve 100% success rate of acquiring the two signals (red and green) corresponding to the break-apart probes described in the product information sheet supplied by the company. The two signals can be clearly seen as two separate red, corresponding to SYT, 18q11.2 and green, corresponding to SYT, 18q11.2 signals and in some instances, the appearance of yellow signals which corresponds to the red and green signal being in close proximity to each other. These findings suggested that Optimised Dako FISH protocol is more suited for use with the tested probe on paraffin embedded tissues in comparison to the Cytocell FISH protocol and can be applied in the diagnostic settings due to the clear appearance of the two signals from the probe that are important in the molecular diagnosis of synovial sarcoma. 

## Discussion

In this study, we have explored two different FISH protocols to optimize the performance of SYT-SSX probe purchased from Cytocell. We have shown that the Optimised Dako FISH protocol is superior to the Cytocell FISH protocol provided by the manufacturer in regards to enabling optimum probe performance. Our finding allows for the establishment of synovial sarcoma FISH diagnosis in our laboratory, which is in conjunction with the latest WHO classification that recommends the inclusion of cytogenetic testing in the diagnosis of soft tissue tumors. Cytogenetic testing, especially FISH is important to enable the targeted diagnosis of soft tissue sarcomas that have overlapping features such as synovial sarcoma, poorly differentiated Ewing sarcoma, solitary fibrous tumour, and malignant peripheral nerve sheath tumour (Lindberg et al., 2015). More importantly, the success of this study will also allow for future inclusion of other FISH testing utilizing Optimised Dako FISH protocol that will expand our capability to diagnose more tumour types. 

The application of FISH on paraffin embedded tissue has not been widely explored due to the technicality of the procedure and also it requires extensive optimizations to ensure quality results that can be used in diagnosis (Ventura et al., 2006).

This study utilizes thin section of tissues, similar to those used in Haematoxylin and Eosin stains that enables both form of analysis to be directly compared. In addition, the use of thicker tissue might require additional treatment steps to attain clear observation of the nucleus. These extra steps might damage the structure of the tissue, leading to difficulties in the analysis of the results. 

Factors such as tissue types, tissue age, tissue thickness and experimental conditions can influence the ability of probes used to access target DNA sequence, which may result in unsuccessful FISH procedure. Pre-treatment conditions are considered as an important step that can determine the success of FISH and thus required strict optimization. The process of pre-treatment is important because steps such as sodium bisulfite washes, trypsin incubation and series of incubations with ethanol ensures the removal of proteins that are not required for the reaction as well as to promote the destruction of protein cross-linking that results from tissue exposure to formalin (Zordan, 2011). 

In this study however, we do not have information of the make-up of the difference solutions provided by the kits, thus we were unable to determine if the differences in materials used in the making of these solutions contributed to the differences in the results observed. The protocols however differ in the preparation of the pre-treatment solution, in which Optimised Dako FISH protocol recommended the incubation of the solution at 97ᵒC for 5 hours prior to tissue incubation, which results in the detection of signals corresponding to the probes used. Based on this observation, we conclude that this step is an extremely important step that prevents temperature fluctuations which result in a proper tissue response to the treatment. It is also plausible that the pretreatment solutions provided by the two manufacturers contain different components that resulted in the different results observed. As pretreatment is considered to be one of the most important steps in FISH (Hicks and Tubbs, 2005), our observation suggest pretreatment solution used in the Optimised Dako FISH protocol may contain components that are more suitable for use in paraffin embedded tissue as well as having better compatibility with SYT-SSX probe used in this particular experiment. 

Proper enzymatic treatment protocol is important to ensure proper probe binding. Bogdanovska-Todorovska et al., (2018) has shown that extended enzymatic treatment resulted in reduction in probe and DAPI signal, whereas inadequate enzymatic digestion can lead to the increase in non-specific signal (Bogdanovska-Todorovska et al., 2018). Optimised Dako FISH protocol emphasizes the importance of enzyme treatment to ensure optimal results. The protocol emphasizes the use of 37ᵒC as the treatment temperature as well as custom enzyme digestion time dependent on the types of tissues as oppose to room temperature and constant enzyme treatment time regardless of tissue types in Cytocell FISH protocol. The temperature used in Optimised Dako FISH protocol has been optimized with the enzyme used while the cell type dependent treatment time ensures optimum enzyme digestion of the tissue (Zordan, 2011). 

The other differences between the two protocols is the use of temperatures for the denaturation and hybridization step. While both protocols claimed to have optimized the temperature, the higher temperature introduced by Optimised Dako FISH protocol might have been one of the factors that positively influenced the results obtained in this study. The higher temperature during the denaturation process might be responsible in combating the formalin-induced tissue resistance to heat, thus ensuring optimal denaturation of DNA and probe accessibility (Zordan, 2011). 

In this short project, we have shown that Optimised Dako FISH protocol overcomes Cytocell FISH protocol in terms of giving positive FISH results. Optimised Dako FISH protocol utilizes Dako kits that emphasizes on prolonged pre-treatment solution prior to slide incubation and custom enzyme incubation timings for different type of tissues. It is also possible that Dako kits might be more suited to be used for the treatment of paraffin embedded tissue as compared to FISH treatment kits from Cytocell. 
